# Comprehensive Overview of Cytokine Interplay in Vitiligo: A Decade of Meta-Analyses Systematically Reviewed

**DOI:** 10.3390/life15050684

**Published:** 2025-04-23

**Authors:** Alessia Paganelli, Cristina Cristofoletti, Francesco Moro, Alessandra Corrente, Laura Colonna, Emanuele Scala, Mauro Picardo

**Affiliations:** 1 Dermatology Unit, Istituto Dermopatico dell′Immacolata-Istituto di Ricovero e Cura a Carattere Scientifico (IDI-IRCCS), via dei Monti di Creta 104, 00167 Rome, Italy; f.moro@idi.it (F.M.); a.corrente@idi.it (A.C.); l.colonna@idi.it (L.C.); m.picardo@idi.it (M.P.); 2 Clinical Trial Center, Istituto Dermopatico dell′Immacolata-Istituto di Ricovero e Cura a Carattere Scientifico (IDI-IRCCS), 00167 Rome, Italy; c.cristofoletti@idi.it; 3 Laboratory of Experimental Immunology, Istituto Dermopatico dell′Immacolata-Istituto di Ricovero e Cura a Carattere Scientifico (IDI-IRCCS), 00167 Rome, Italy; emanuele.scala@idi.it

**Keywords:** vitiligo, cytokines, TNF-α, INF-γ, IL-17, immunology, dermatology, meta-analysis, chemokine, interleukin

## Abstract

(1) Background: Vitiligo is an autoimmune skin disorder characterized by melanocyte destruction. Despite metabolic disturbances and oxidative stress also playing a key role in its pathogenesis, accumulating evidence highlights a prominent role for cytokine dysregulation. (2) Methods: A systematic search was conducted to identify meta-analyses published in the last decade that investigated cytokine involvement in vitiligo. (3) Results: Based on predefined inclusion criteria, nine meta-analyses were retrieved and reviewed. The findings confirm a central role for interferon-gamma (IFN-γ) in vitiligo pathogenesis, although recent meta-analyses suggest that IFN-γ gene polymorphisms are more broadly associated with autoimmunity rather than being vitiligo-specific. Elevated interleukin-17 (IL-17) levels have been consistently reported in vitiligo patients, supporting its contribution to immune-mediated melanocyte destruction. Regulatory T cell dysfunction appears to play a crucial role in disease progression. Additionally, TNF-α-308 G/A polymorphism has been linked to a genetic susceptibility to vitiligo, particularly in specific populations, reinforcing the role of TNF-α in immune dysregulation. Lastly, chemokines involved in immune cell recruitment to melanocytes further illustrate the complex inflammatory network underlying the disease. (4) Conclusions: This systematic review consolidates evidence from a decade of meta-analyses, underscoring the significance of cytokine dysregulation in vitiligo and highlighting potential therapeutic targets.

## 1. Introduction

Vitiligo is a chronic skin disorder characterized by well-defined areas of depigmentation [1]. Current estimates suggest a global prevalence of approximately 0.5% to 2% [2,3]. Although the average age of onset falls between 20 and 30 years, the disease can also manifest in both children and older adults. Regarding sex distribution, most studies report an equal occurrence in males and females [4]. Large-scale epidemiological analyses indicate that up to 20% of patients have at least one affected family member, with familial cases typically presenting with an earlier onset [5].

While vitiligo is not a life-threatening condition, it significantly impacts patients’ quality of life, affecting self-esteem, social interactions, and professional relationships [6,7]. The burden of the disease is particularly pronounced in women, adolescents, and individuals with psychiatric conditions [8].

The pathogenesis of vitiligo is thought to involve multiple mechanisms, including genetic predisposition, immune system dysregulation, oxidative stress, environmental triggers, and metabolic disturbances [9]. Notably, approximately 10–20% of vitiligo patients develop coexisting autoimmune or autoinflammatory conditions, such as autoimmune thyroiditis, type 1 diabetes (T1D), systemic lupus erythematosus, Addison’s disease, alopecia areata, or lichen sclerosus [10,11,12]. However, recent studies emphasize that vitiligo patients also frequently exhibit metabolic disorders beyond T1D, such as insulin resistance, type 2 diabetes, dyslipidemia, and metabolic syndrome [13,14]. Consequently, individuals with vitiligo may face an increased risk of cardiovascular complications compared to the general population [15,16].

Metabolic dysfunctions in vitiligo have been linked to imbalances in pro-inflammatory factor secretion, reduced oxidative stress, and impaired antioxidant defense mechanisms [17,18,19]. Nevertheless, the precise mechanisms underlying these alterations remain unclear.

Despite metabolic alterations possibly representing the *primum movens* in the pathogenesis of vitiligo [20], it is doubtful that cytokine imbalance represents a key feature of the disease.

The aim of the present work is to provide a comprehensive overview of the published literature in this setting, through a systematic literature review of available meta-analyses.

## 2. Materials and Methods

**Figure 1:**
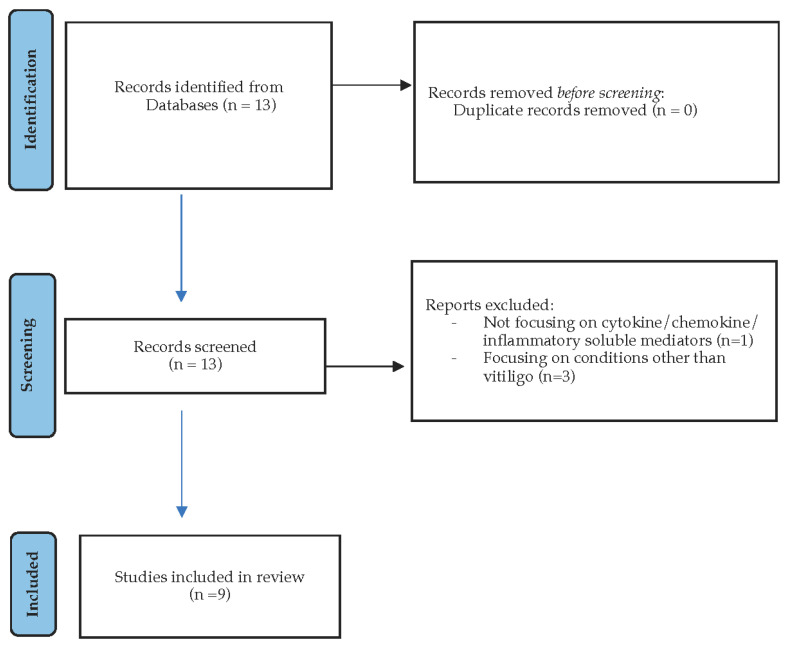
PRISMA flow diagram for systematic reviews indicating the search strategy used in our study.

This systematic review was performed in compliance with the PRISMA guidelines. A flow chart diagram is presented in Figure 1.

### 2.1. Search Strategy

A search was conducted in the MEDLINE and Scopus electronic databases from 2015 to present (up to February 2025). The detailed search strategy for MEDLINE (PubMed) used the following terms: ‘vitiligo’ [Title/Abstract]) AND ‘cytokines’ [Title/Abstract]. The terms were adapted for the other database as appropriate. All the major journals were indexed. Only journal articles were taken into consideration, while books and book chapters were excluded. Articles without a full text electronically available and/or English translation were also excluded. Only meta-analyses were taken into consideration. Articles not specifically focusing on the role of cytokines in vitiligo were excluded.

### 2.2. Study Population—Selection

The following PICO (Population, Intervention or exposure, Comparison, Outcome) elements were applied as inclusion criteria for the systematic review: (i) Population: patients affected by vitiligo and/or in vitro models of vitiligo. (ii) Intervention: use of metanalyses to assess and validate cytokine dysregulation. (iii) Comparator: role of cytokines in other dermatological pathological and physiological conditions. (iv) Outcome: identification of the most important cytokine pathways dysregulated in the setting of vitiligo and the identification of potential new therapeutic targets. No general risk-of-bias assessment tool could be used due to the heterogeneity of the meta-annalyses considered (for details on risk of bias assessment performed in each included meta-analysis, refer to the studies listed in Table 1).

### 2.3. Data Extraction

For studies fulfilling the inclusion criteria, three independent reviewers (E.S., F.M., and A.P.) extracted data in a standardized and predefined form. Disagreements were resolved by a fourth reviewer (C.C.). The following data were collected for each paper: first author, year of publication, cytokine(s) of interest, type of study (human, animal, in vitro), main outcomes, therapeutic implications. All the co-authors reviewed the final table and figures. The number of papers published per year was also calculated to get an idea of the field’s growth.

## 3. Results and Discussion

**Figure 2:**
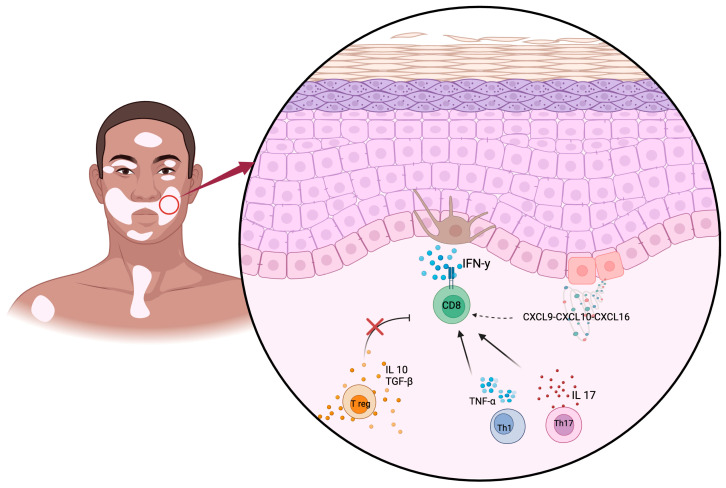
Key immunological pathogenetic mechanisms in vitiligo. Melanocyte destruction is mostly mediated by IFN-γ secreting CD8+ CTLs, recruited in situ by specific chemokines. Treg dysfunction and increased concentration of other pro-inflammatory mediators also contribute to disease development. Created in BioRender Version 04.

In total, nine papers matched our search criteria and were therefore considered in the present review (Table 1). An overview of the main pathogenetic pathways that emerged from our work is summarized in Figure 2.

### 3.1. IFN-γ: A Key Player in Melanocyte Destruction

Cytotoxic T lymphocytes (CTLs) are the major reason for melanocyte depletion in vitiligo, with Fas–FasL pathway activation being a crucial molecular mechanism [30]. Notably, interferon-gamma (IFN-γ) is a pro-inflammatory cytokine produced by T helper 1 (Th1) cells, natural killer (NK) cells, and CTLs, and it plays a critical role in vitiligo pathogenesis and in CTL-mediated melanocyte destruction in depigmented areas [31,32]. Not surprisingly, elevated serum concentrations of IFN-γ have been detected in patients with active vitiligo, correlating with disease progression and activity [33]. However, the exact role of INF-y in Fas-mediated apoptosis has yet to be clarified [32,34,35].

In more detail, the signaling axis involving IFN-γ, C-X-C motif chemokine ligands (CXCL9/10), and the C-X-C motif chemokine receptor (CXCR3) has been demonstrated to induce the recruitment of autoreactive CD8+ T cells, which ultimately induce melanocyte detachment and apoptosis [36].

Other studies have found IFN-γ to induce apoptosis in human melanocytes by activating the JAK1/STAT1 (Janus kinase/signal transducers and activators of transcription-1) signaling pathway, alongside increasing the expression levels of Bax, Bak, and cleaved caspase-3 and decreasing the expression levels of Bcl-2 [37].

IFN-γ binds to two cell surface receptors, IFNGR1 and IFNGR2, associated with JAK1 and JAK2, respectively. This mechanism leads to the phosphorylation of STAT proteins, which then enter the nucleus, activate gene transcription, and upregulate several factors involved in the recruitment and activation of immune cells [38,39]. JAK inhibition therefore reduces IFN-γ-mediated immune activation and prevents immune-mediated melanocyte destruction, facilitating melanocyte survival and allowing skin repigmentation [40]. Recently JAK inhibitors have demonstrated clinical efficacy in the treatment of vitiligo [41,42]. Clinical trials have demonstrated improvements, especially in early or localized vitiligo, with benefits such as repigmentation and reduced autoimmunity [43,44,45]. Interestingly, JAK inhibitor therapy has been used to treat several immune-mediated skin conditions, including psoriasis and vitiligo [46,47]. However, while the blockage of the JAK/STAT axis in psoriasis just prevents the cytokine-mediated activation of Th17 cells, in vitiligo direct interference with IFN-γ-mediated melanocyte apoptosis induced by CTL also occurs.

Moreover, IFN-γ not only affects the immune system but also directly interferes with melanogenesis [48,49].

Furthermore, IFN-γ is known to promote the release of pro-inflammatory cytokines, such as tumor necrosis factor-alpha (TNF-α), interleukin-6 (IL-6), and interleukin-1β (IL-1β), which not only contribute to the local inflammatory microenvironment within lesional skin but also correlate with systemic inflammation in vitiligo patients [50,51,52]. Of note, IFN-γ acts synergistically with TNF-α in upregulating the expression of Fas on melanocytes; however, TNF-α and IFN-γ take concerted action in inducing the upregulation of anti-apoptotic genes, such as c-IAP2, c-FLIP and MCL1 [53]. These data could at least partially explain the increased risk of vitiligo following anti-TNF therapy [54].

Variants in genes involved in IFN-γ signaling, such as IFNGR1 (the receptor for IFN-γ) and STAT1 (a downstream transcription factor), have been associated with an increased risk of vitiligo, suggesting a genetic predisposition that enhances the inflammatory response in affected individuals [55].

The functional polymorphism rs2430561 in the IFN-γ gene is known to enhance IFN-γ expression, and several studies have linked the IFN-γ +874 T/A polymorphism to the development of various autoimmune diseases. A study on 175 subjects (85 cases, 90 controls) indicated that such a genetic signature (T allele, TT genotype) could be associated with more severe forms of the disease [56]. The authors reported a 1.7-fold increased risk of vitiligo in the TA haplotype. The same study also confirmed elevated serum concentrations of IFN-γ and other pro-inflammatory cytokines in vitiligo patients compared with the control group. Interesting, even higher cytokine levels were detected in the presence of active disease compared to stable vitiligo.

However, conflicting data have been published so far in this setting, with other reports not having confirmed such an association. Our search identified only one meta-analysis aimed at assessing whether the IFN-γ +874 T/A polymorphism really influences the risk of autoimmune diseases [28]. Eighteen studies, including 2952 patients and 3832 controls, were analyzed. Overall, the meta-analysis showed no significant association between the IFN-γ +874 T allele and autoimmune diseases in general (odds ratio [OR] = 1.023, 95% confidence interval [CI] = 0.894–1.171, *p* = 0.738). However, data stratification by ethnicity provided new insights, indicating such a polymorphism to possibly confer a higher risk of developing autoimmune disorders in selected ethnic and genetic backgrounds.

Even more importantly, stratification according to disease type showed a significant association between the IFN-γ +874 T allele and idiopathic thrombocytopenic purpura (ITP) and systemic lupus erythematosus (SLE), but no significant associations were found for vasculitis, vitiligo, or autoimmune thyroid disease. Taken together, these data suggest that the IFN-γ +874 T/A polymorphism is associated with an increased risk of autoimmune diseases, particularly ITP and SLE, but other mechanisms are probably responsible for IFN-γ overproduction in the setting of vitiligo.

Likewise, the role of IFN-γ polymorphisms has also been investigated in the setting of psoriasis [57]. However, only one Polish study reported an increased susceptibility to psoriasis in patients with specific genetic variants in the IFN-γ promoter [58]. Nonetheless, significant bidirectional associations between psoriasis and vitiligo exist: psoriasis patients are 2.29 times more likely to have vitiligo, and vitiligo patients have a 3.43-fold increased risk of developing psoriasis [59]. A possible explanation for this relationship is shared inflammasome-related genetic variants and a common susceptibility locus in the major histocompatibility complex [60,61,62]. Furthermore, overlapping immune pathways may play a role in the coexistence of both diseases [59]. A patient treated with IFN-α for hepatitis B was reported to have developed both vitiligo and psoriasis simultaneously [63]. IFN-α, mostly produced by plasmacytoid dendritic cells, may contribute to the development of both conditions through Th1- and Th17-mediated autoimmune inflammation of the skin [59].

As a direct consequence of the identification of IFN-γ as a crucial inflammatory mediator in vitiligo pathogenesis, promising data are currently emerging from clinical trials aimed at investigating the effectiveness of monoclonal antibodies targeting IFN-γ or its receptor in modulating the immune response and halting disease progression [31,56,64,65].

In summary, IFN-γ is a pivotal cytokine in the pathogenesis of vitiligo, promoting melanocyte apoptosis and depletion either directly or through immune-mediated mechanisms. Its involvement in both local melanocyte dysfunction and systemic immune dysregulation underscores its central role in vitiligo progression, making it both a promising immunological marker and a key target for novel therapeutic strategies.

### 3.2. Chemokines

Chemokines are key signaling proteins that guide immune cells, such as T cells, monocytes, and dendritic cells, to sites of inflammation or injury [66,67]. In vitiligo, chemokines—especially those released by melanocytes—play a pivotal role in disease pathogenesis [68]. Research has shown that chemokines associated with a Th1-dominant immune response are elevated in the skin of vitiligo patients, particularly during active phases of the disease [36,69]. Notably, CXCL10 and CXCL9 are critical mediators, attracting immune cells to the skin and amplifying the autoimmune attack on melanocytes [36,69,70]. A recent meta-analysis, however, also highlighted elevated levels of CCL5 (CC motif chemokine ligand 5), CXCL8, CXCL12, and CXCL16, emphasizing the complexity of the immune landscape in vitiligo [71].

An elevated CXCL10 concentration in the epidermis is known to enhance CD8+ T cell infiltration, driving inflammation in active vitiligo lesions [36]. Of note, vitiligo melanocytes produce significantly more CXCL10 than healthy melanocytes, making them more susceptible to immune attacks [72]. Furthermore, CXCL10 recruits NK cells and activated monocytes, amplifying the inflammatory response [73,74]. Similarly, CXCL9 acts as a potent chemoattractant for both CD4+ Th1 and CD8+ T cells, promoting Th1 polarization and the production of pro-inflammatory cytokines like IFN-γ, which in turn enhances CXCL10 and CXCL9 expression, creating a feedback loop that sustains inflammation [36,75].

CCL5 has been demonstrated to be increased in vitiligo [76], with RNA analysis showing a 23.5-fold upregulation of such a chemokine in cultured vitiligo melanocytes [77]. However, the relationship between CCL5 levels and disease activity and/or severity remains unclear. In fact, while two studies found no correlation with disease severity, one report found an increased CCL5 concentration (1.23-fold higher) in active vitiligo [29]. CCL5 is typically associated with Th1 responses, but it may also influence Th2 pathways [78,79]. Though Th1 and Th2 responses were once considered mutually exclusive, recent findings suggest that Th2-related cytokines can cooperate with Th1 cytokines to enhance the migration, generation, and activation of memory CD8+ T cells in vitiligo skin [79].

CCL5 binds several receptors, with the highest affinity for its receptor CCR5 (C-C chemokine receptor type 5) on immune cells [80]. However, the activation of the CCL5-CCR5 axis is not limited to lesional CTLs: CCR5 expression is upregulated on regulatory T cells (Tregs) in vitiligo [29], suggesting (dysfunctional) Tregs could possibly contribute to skin depigmentation [81]. As such, targeting the CCL5-CCR5 axis could provide new therapeutic options for vitiligo.

CXCL8 (IL-8) is a proinflammatory cytokine produced by various cell types—including melanocytes—that recruits neutrophils via CXCR1 and CXCR2 [77,82]. Of note, CXCR1+ CD8+ T cells responding to CXCL8 are mostly involved in cytotoxicity and early migration [82]. The recruitment of neutrophils by CXCL8 is crucial in the vitiligo-specific inflammatory response and may also contribute in inducing an IL-17-driven Th17 response [83]. This highlights potential therapeutic strategies aimed at reducing inflammation and restoring pigmentation.

CXCL12 (SDF-1), which recruits lymphocytes and macrophages through its receptor CXCR4, influences both immune cell attraction and melanocyte survival in vitiligo [38]. Noteworthily, cultured melanocytes produce this chemokine in response to lipopolysaccharides (LPSs), with RNA analysis showing a 55.3-fold upregulation in vitiligo melanocytes compared to healthy controls [77]. Moreover, CXCL12 plays a role in the migration and differentiation of melanocyte precursors, which can help with repigmentation [29]. Some authors also postulated that CXCL12 may contribute to hair graying by facilitating the migration of melanocyte precursors away from the hair bulb, thereby disrupting normal pigmentation processes [84,85].

CXCL16, primarily produced by keratinocytes in response to reactive oxygen species (ROS), recruits T lymphocytes, particularly CD8+ T cells and NK cells, through its receptor CXCR6 [86,87]. In vitiligo, CXCL16 promotes the infiltration of CTLs, exacerbating melanocyte destruction and inflammation [88]. An elevated serum CXCL16 concentration has also been reported in vitiligo patients, confirming its implication in disease progression [29].

However, several other chemokines have been investigated in the setting of vitiligo [76,89]. With regards to CCL3 production, contrasting data have been published so far, with some evidence indicating an increase ([76,90]), and other studies suggesting decreased levels in vitiligo [91]. CCL3 is produced by a variety of hematopoietic and non-hematopoietic cells and recruits macrophages, eosinophils, and lymphocytes via the CCR1 or CCR5 receptor, with preferential activity on activated CD8+ T cells [92].

As research into chemokines in vitiligo progresses, further investigation of chemokine signaling pathways will be essential for developing effective treatments aimed at restoring pigmentation and normalizing immune function in this chronic condition.

### 3.3. IL-17: A Controversial Player in Vitiligo Pathogenesis and Treatment

Several studies have demonstrated a correlation between IL-17 levels and melanocyte function, suggesting its potential involvement in vitiligo pathogenesis. With regards to preclinical studies, IL-17 secretion has been found to be associated with depigmentation in murine models [83]. IL-17 exposure leads to a reduction in melanocyte count, melanin content, and tyrosinase levels [93]. Bhardwaj et al. demonstrated that blocking the IL-17A receptor could promote melanocyte survival and enhance melanin production in vitro, therefore suggesting a potential therapeutic role for anti-IL-17 in the setting of vitiligo [93]. Not surprisingly, elevated IL-17 mRNA expression levels and increased Th17 cell frequencies have been described both in blood and lesional skin from vitiligo patients [94].

A meta-analysis conducted by Acharya et al. reviewed 11 case–control studies assessing blood IL-17 concentrations in vitiligo patients compared to healthy subjects [21]. The analysis confirmed a significant increase in circulating IL-17 levels among individuals with vitiligo. Furthermore, three of these studies examined IL-17 levels in lesional skin: in line with previously mentioned data, the IL-17 concentration was consistently higher in lesional tissue compared to healthy skin. These findings reinforce the notion that the IL-17 pathway is upregulated in vitiligo and may play a role in disease progression. Such an hypothesis is further supported by the observation of reduced levels of IL-17, as well as IFN- γ, in hyperpigmentary disorders [95].

However, emerging clinical evidence suggests that IL-17 inhibition may not be a therapeutic approach, as a IL-17A blockade has been associated with a paradoxical worsening or induction of vitiligo in some patients [21].

A case reported by Su et al. described a 72-year-old woman with chronic plaque psoriasis and psoriatic arthritis who developed depigmented patches on her face after four months of treatment with Ixekizumab, a monoclonal antibody targeting IL-17A. Upon discontinuation of Ixekizumab and initiation of cyclosporine, the patient experienced a significant repigmentation, suggesting that broad immunosuppression rather than a IL-17 blockade may be necessary to prevent melanocyte loss [96].

Similarly, Pirro et al. described the case of a 48-year-old man who developed vitiligo on his legs, hands, and feet after three months of Ixekizumab therapy for psoriasis. The patient received topical 0.1% tacrolimus for eight weeks, without discontinuing Ixekizumab. Partial repigmentation was achieved after four months, suggesting that local immune modulation may provide some benefit without requiring systemic therapy [97].

Further evidence comes from a pilot study by Speeckaert et al. [29], which investigated the effects of Secukinumab, another IL-17A inhibitor, in patients with active non-segmental vitiligo: most patients developed new lesions, leading to the premature termination of the study. These findings indicate that IL-17 inhibition may paradoxically exacerbate vitiligo.

Méry-Bossard and collaborators also reported additional cases of de novo vitiligo and the worsening of pre-existing vitiligo in patients receiving biologic therapies for chronic inflammatory diseases, including IL-17 inhibitors [98].

Taken together, these clinical reports highlight a paradox: although IL-17 is elevated in vitiligo and contributes to inflammation, its inhibition does not appear to confer therapeutic benefits and may instead lead to the disease’s exacerbation. Therefore, targeting IL-17 alone may be insufficient or even counterproductive.

### 3.4. Tumor Necrosis Factor Alpha (TNF-α) and Its Role in Vitiligo

TNF-α is produced primarily by immune cells, particularly activated macrophages and T cells. It plays a pivotal role in immune system regulation, mediating inflammatory responses, and apoptosis [99]. TNF-α has been implicated in the pathogenesis of a variety of autoimmune diseases, with recent studies having highlighting its involvement in vitiligo as well.

Elevated levels of TNF-α have been detected in the serum and lesional skin of vitiligo patients, suggesting its contributory role in melanocyte destruction [100,101]. The exact mechanism by which TNF-α contributes to vitiligo is not fully elucidated; however, it has been hypothesized that increased TNF-α concentrations may lead to melanocyte apoptosis or impair their function, thereby contributing to depigmentation [90,102].

First of all, TNF-α has been shown to enhance the activity of CD8+ CTLs, involved in the attack on and destruction of melanocytes [103]. Additionally, TNF-α is implicated in dendritic-cell activation, which plays a crucial role in initiating the immune response in vitiligo lesions [104].

Moreover, TNF-α interacts with its receptors (TNFR1 and TNFR2) on melanocytes, leading to apoptotic cell death through various mechanisms, including the activation of the caspase cascade and the production of reactive oxygen species (ROS) [82].

Lastly, TNF-α stimulates the production of other cytokines, including IFN-γ, which further promotes the autoimmune response against melanocytes [101,102].

Genetic factors play a significant role in the regulation of TNF-α in vitiligo. Several studies have investigated the association between specific genetic polymorphisms in the TNF-α gene and susceptibility to vitiligo [24,26,105,106,107]. The TNF-α gene is located on chromosome 6p21.3. One of the most extensively studied polymorphisms is the TNF-α -308G/A single nucleotide polymorphism (SNP) [108]. Such a guanine (G) to adenine (A) substitution in the promoter region of the TNF-α gene potentially influences its transcriptional activity and, consequently, production [108]. For instance, certain TNF gene variants have been found to be more prevalent in patients with vitiligo, suggesting a genetic base for higher TNF-α levels [108]. Additionally, environmental factors such as UV radiation, oxidative stress, and infections may trigger the release of TNF-α, contributing to the onset or exacerbation of vitiligo in genetically susceptible individuals [109,110].

Several meta-analyses have investigated the association between TNF-α gene polymorphisms and susceptibility to vitiligo, with a particular focus on the -308 G>A variant.

A meta-analysis performed by Wu et al., for example, concluded that the TNF-α -308G/A polymorphism might not be significantly associated with an increased risk of vitiligo. However, the authors acknowledged that, due to the limited number of studies and sample sizes, further research is needed to confirm these findings [23].

Nie et al. also investigated the association between the TNF-α-308G/A polymorphism and vitiligo. The study synthesized data from six case–control studies, encompassing 1391 vitiligo patients and 2455 healthy controls. The analysis revealed no significant differences in the distribution of TNF-α-308G/A genotypes between vitiligo patients and controls across various genetic models, emphasizing the need for further data to confirm a potential association between such a genetic variant and the development of vitiligo [25].

A comprehensive meta-analysis published in 2021 by Laddha et al. analyzed 11 studies with 2199 vitiligo patients and 3083 controls. The results indicated that the TNF-α -308A allele and AA, GA, and AA + GA genotypes were associated with an increased risk of vitiligo in the overall population, despite the presence of regional and ethnical variability [27].

A study published in 2022 by Giri PS et al. suggested that the ‘A’ allele could be associated with an increased risk of vitiligo, in particularly among Asian, Middle Eastern, and Egyptian groups, therefore confirming the potential role of the TNFA-308 (G > A) SNP in vitiligo pathogenesis across diverse ethnicities [27].

More recently, Dutta et al. in 2024 focused their attention on the Asian population. This comprehensive meta-analysis analyzed 31 case–control studies encompassing eleven SNPs. The study identified the rs1800629G>A variant upstream of the TNF-α gene as significantly associated with an increased vitiligo risk in additive, dominant, and recessive models. In silico analyses suggested that this variant has a regulatory potential, possibly contributing to vitiligo’s pathogenesis in the examined population [26].

Taken together, these findings emphasize the need for population-specific research in order to develop targeted therapeutic strategies and personalized medicine approaches.

While the potential pathogenetic role of TNF-α in vitiligo is still debated, emerging evidence points at anti-TNF biologics as potential triggers of vitiligo. In fact, several reports have associated the de novo onset of vitiligo with the use of anti-TNF-α agents [54,100]. The potential mechanism by which anti-TNF-α agents may induce vitiligo involves activating alternative cytokine pathways (such as type I interferons) and suppressing Treg production and activation. This results in the failure to suppress the T cell response against melanocytes [98,111].

However, other reports suggest that TNF-α inhibition is more likely to result in cutaneous hyperpigmentation rather than hypo- or depigmentation [112]. In fact, high concentrations of TNF-α seem to be possibly associated with reduced tyrosinase activity, therefore leaving room for anti-TNF biologic drugs in the treatment of vitiligo [112]. When considering anti-TNF-α therapy in patients with de novo vitiligo, some studies suggest that Etanercept may be the preferred agent to minimize the risk of developing paradoxical vitiligo reactions and block disease progression [100].

Given the complex and multifactorial nature of vitiligo, further research is needed to better understand the precise mechanisms by which TNF-α contributes to vitiligo, and a comprehensive understanding of TNF-α’s interactions with other cytokines and immune pathways is essential before translating these findings into clinical practice.

### 3.5. Immune-Tolerance Disruption

As with other autoimmune disorders, vitiligo has been described as possibly being associated with defective immune tolerance. In particular, several reports have been published so far on a possible decrease in circulating CD4(+) CD25(+) Tregs and Treg-related cytokines in vitiligo patients [113].

Interleukin-10 (IL-10) is an anti-inflammatory cytokine essential for immune regulation, as it suppresses excessive immune responses and helps maintain immune homeostasis. It is primarily produced by regulatory T cells (Tregs), macrophages, dendritic cells, B cells, and certain T helper cell subsets, functioning to inhibit pro-inflammatory cytokine production and antigen presentation [114,115,116]. Reduced IL-10 concentrations may contribute to the breakdown of immune tolerance in vitiligo, leading to the destruction of melanocytes and the progression of depigmentation [117,118].

TGF-β is a renowned pro-fibrotic and immune-suppressive cytokine secreted by a wide range of cell sources, including Tregs, Tr1 cells, fibroblasts, mesenchymal stromal cells, platelets, dendritic cells, and macrophages [119,120,121,122]. In particular, the TGF-β concentration has been found to be significantly decreased both in serum and PBMC supernatants from active vitiligo compared to the stable disease [113]. Similarly, the same study found TGF-β to negatively correlate with affected BSA (body surface area) [113]. As for the cellular and molecular mechanisms leading to a TGF-β decrease in vitiligo, stromal fibroblasts seem to be particularly susceptible to CD8+ T cell-secreted IFN-γ, with a subsequent downregulation of early growth response 1 (EGR1) [101,123], ultimately leading to a TGF-β1 deficiency.

However, conflicting data have been published so far on TGF-β‘s involvement in vitiligo’s pathogenesis, with other studies indicating increased serum levels of TGF-β in non-segmental vitiligo [124]. Moreover, serum TGF-β1 level swere also proposed to directly correlate with Th17 cell frequencies and BSA involvement, underscoring a controversial role for TGF-β in vitiligo’s pathogenesis [124].

A research protocol evaluating skin samples obtained from both lesional and non-lesional areas of 30 patients with nonsegmental vitiligo, compared to normal skin, confirmed significant alterations in immune-suppressive pathways [125]. In more detail, not only was FOXP3 expression found to be decreased in lesional skin, but also the IL-10 concentration was significantly reduced. In contrast, TGF-β expression was elevated in vitiligo compared to controls. However, its levels were lower in lesional than in non-lesional skin, again underscoring its ambiguous role in vitiligo’s pathogenesis. These findings indicate that Treg cell dysfunction and IL-10 deficiency may contribute to the autoimmune mechanisms underlying vitiligo.

A meta-analysis was recently performed to rule out the role of Tregs and Treg-associated cytokines in the setting of vitiligo. A total of 30 studies, including 1223 vitiligo patients and 1109 controls, were analyzed [22]. A significantly lower frequency of Treg cells was confirmed in vitiligo patients compared to healthy controls. Notably, the ability of Tregs to suppress CD8+ T cells was also markedly reduced.

Furthermore, FOXP3, a crucial transcription factor for Tregs, was significantly downregulated in both the blood and skin of vitiligo patients (*p* < 0.00001). [22] Treg frequency and FOXP3 expression were found to inversely correlate with disease activity. Even more importantly, the results of this meta-analysis confirmed key Treg-associated suppressive cytokines, IL-10 and TGF-β, to be significantly reduced in vitiligo patients [22]. IL-10 expression, in particular, was found to increase significantly after treatment both in clinical and pre-clinical (murine) studies.

The current literature underscores the critical role of Tregs’ dysfunction in vitiligo’s pathogenesis, highlighting their reduced frequency, impaired function, and diminished cytokine production. Future clinical trials focusing on Treg-based therapeutic interventions could pave the way for novel, more effective treatments for vitiligo.

## 4. Conclusions

Our paper systematically assesses the available meta-analyses on cytokines in vitiligo and therefore represents an “umbrella review”, providing a high-level synthesis of the existing evidence with a broad, systematic perspective [126]. However, this type of study has several intrinsic limitations, the main being the heterogeneity among meta-analyses in terms of study design, inclusion criteria, statistical methods, and population characteristics, making direct comparisons challenging. Moreover, any biases or methodological flaws in the original studies may be compounded. Unlike systematic reviews of primary studies, umbrella reviews also lack access to raw patient data, limiting the ability to explore subgroup analyses or adjust for confounders. Additionally, the relatively low number of available meta-analyses limits the generalizability of the findings, highlighting the need for further large-scale, high-quality studies to refine our understanding of cytokine involvement in vitiligo’s pathogenesis.

Despite the existing limitations, this systematic review underscores the critical role of cytokines in vitiligo’s pathogenesis. While a central role for IFN-γ has been confirmed in this setting, recent meta-analyses found IFN-γ genic polymorphisms to be more likely linked to autoimmunity in general rather than being vitiligo-specific. Elevated IL-17 levels have been consistently reported in vitiligo patients, highlighting its contribution to immune-mediated melanocyte destruction. Similarly, regulatory T cell dysfunction appears to play a significant role in disease progression. The role of TNF-α in vitiligo is quite controversial, but meta-analyses suggest the genetic predisposition to vitiligo in specific populations to be associated with specific *TNF-α* polymorphisms, reinforcing the role of TNF-α in disease susceptibility and immune dysregulation. Last but not least, chemokines, particularly those involved in immune cell recruitment to melanocytes, further illustrate the complex inflammatory landscape of the disease.

Although there is substantial evidence supporting the significant role of cytokine dysregulation in the pathogenesis of vitiligo, it remains uncertain whether these alterations are truly specific to vitiligo or more broadly associated with autoimmune conditions in general. Vitiligo patients frequently present with autoimmune comorbidities, which may influence the concentrations of cytokines and chemokines evaluated in studies [10,127,128,129]. Autoimmune thyroid diseases (e.g., Hashimoto’s thyroiditis and Graves’ disease) are among the most common comorbidities and are typically characterized by the overproduction of IFN-γ and TNF-α [130,131]. Other comorbid disorders include type 1 diabetes, associated with IL-17 and IL-6 dysregulation, and rheumatoid arthritis, typically characterized by TNF-α and IL-1β elevation [11,132]. Studies suggest that vitiligo patients may also have an increased risk of developing celiac disease due to shared autoimmune mechanisms [133,134]; indeed, celiac disease is characterized by an elevated concentration of IL-2, IL-8, and IL-10 [134,135]. Also, rarer concomitant diseases, such as SLE and inflammatory bowel diseases (IBDs), may contribute to increased circulating levels of pro-inflammatory cytokines [136,137]. Of note, vitiligo is often accompanied by other cutaneous immune-mediated disorders, like alopecia areata (associated with an IFN-γ and IL-15 signature) [138,139], atopic dermatitis (typically characterized by an overproduction of Th2-related cytokines, including IL-4, IL-13, and IL-31) [140,141], and psoriasis (driven by IL-17, IL-23, and TNF-α) [142,143,144]. All these concomitant conditions further complicate the immunological landscape of vitiligo, potentially influencing the cytokine profiles in affected individuals.

Overall, these findings consolidate the significance of cytokine dysregulation in vitiligo and highlight potential therapeutic targets for future interventions.

## Figures and Tables

**Table 1:** Main results retrieved through our search. The presence of commercially available drugs targeting crucial pathogenetic cytokines has also been mentioned in the right column. NA: not available; IL: interleukin; TGF: tumor growth factor; TNF: tumor necrosis factor; IFN: interferon; FOXP3: Forkhead box P3; CXCL: C-X-C motif chemokine ligands; CCL: CC motif chemokine ligand; Th1: T helper 1 cells.

Ref.	Year	1st Author	Implicated Cytokine/Chemokine	Main Findings	Available Drugs
[21]	2020	Acharya P	IL-17		Secukinumab, Ixekizumab, Bimekizumab, Brodalumab
[22]	2022	Giri PS	IL-10, TGF-β	reduction of suppressive molecules (FOXP3, IL-10, and TGF-β)	NA
[23]	2015	Wu D	TNF-α	TNF-α-308 G/A polymorphism is not a genetic risk factor for vitiligo	Infliximab, Etanercept, Adalimumab, Golimumab, Certolizumab pegol
[24]	2015	Lee YH		TNF-α -308 A/G polymorphism may be a significant risk factor for vitiligo in Middle Eastern populations	
[25]	2015	Nie G		TNF-α-308G/A polymorphism may not be associated with vitiligo risk	
[26]	2024	Dutta T		TNF-α gene: rs1800629 was found to be associated with vitiligo risk in Asian population	
[27]	2022	Giri PS		TNFA-308(G > A) SNP and vitiligo susceptibility	
[28]	2016	Lee YH	IFN-γ	no association between IFN-γ +874 T/A polymorphism and vitiligo	NA
[29]	2023	Speeckaert R		blood CCL5, CXCL8, CXCL12, and CXCL16 levels were significantly elevated (Th1 response)	NA

## Data Availability

Data are available from the authors upon request.
